# Effect of low and high HDL-C levels on the prognosis of lupus nephritis patients: a prospective cohort study

**DOI:** 10.1186/s12944-017-0622-3

**Published:** 2017-12-06

**Authors:** Peiran Yin, Ying Zhou, Bin Li, Lingyao Hong, Wei Chen, Xueqing Yu

**Affiliations:** 1 Department of Nephrology, The First Affiliated Hospital, Sun Yat-sen University, Guangzhou, Guangdong 510080 China; 2 Key Laboratory of Nephrology, Ministry of Health and Guangdong Province, Guangzhou, Guangdong 510080 China; 3 Guangdong Medical University, Zhanjiang, Guangdong 524023 China; 4 Clinical Research Center, The First Affiliated Hospital, Sun Yat-sen University, Guangzhou, Guangdong 510080 China

**Keywords:** Lupus nephritis, HDL-c, Prognosis

## Abstract

**Background:**

Few data has been available on the effect of serum HDL-C levels on the prognosis of lupus nephritis (LN) patients. The present study therefore aimed to explore the effect of serum HDL-C levels on LN patients.

**Methods:**

We included 775 patients with follow-up information registered in an LN database between 1 January 2006 and 31 December 2011. The patients were divided into groups with low, intermediate and high HDL-C, according to NCEP ATPIII criteria. Cox regression analyses were used to explore the effects of HDL-C levels on end-stage renal disease (ESRD), all-cause mortality and cardiovascular disease (CVD) mortality.

**Results:**

During a median follow-up of 56 months (3–206 months), 71 (9.2%) had ESRD. 84 (10.8%) deaths occurred, 17 (20.2%) of which were due to CVD. There was no statistically significant association of HDL-C category or continuous HDL-C levels with ESRD in the total cohort, but in subgroup analyses by eGFR, with each 0.1 mmol/L increase in HDL-C level, adjusted HRs for ESRD were 0.92 (95% CI: 0.83–1.04, *P* = 0.173) for eGFR ≥60 ml/min/1.73m^2^ and 1.11 (95% CI: 1.01–1.23, *P* = 0.036) for eGFR <60 ml/min/1.73m^2^. The effect of the interaction between eGFR category and serum HDL-C level on ESRD was statistically significant (β = −1.738, *P* = 0.005). Low HDL-C was associated with all-cause mortality (HR = 2.16, 95% CI: 1.06–4.40, *P* = 0.033) with intermediate HDL-C as reference category after adjusting for several variables.

**Conclusions:**

Our results demonstrate that high HDL-C levels were associated with increased risk of ESRD in LN patients with advanced renal dysfunction. While low HDL-C levels were associated with increased risk of all-cause mortality in LN patients.

**Trial registration:**

ClinicalTrials.gov Identifier: NCT03001973, 22 December 2016 retrospectively registered.

**Electronic supplementary material:**

The online version of this article (10.1186/s12944-017-0622-3) contains supplementary material, which is available to authorized users.

## Background

Lupus nephritis (LN) is an immune complex glomerulonephritis that develops as one of the most serious complication of systemic lupus erythematosus (SLE) [1]. Asian SLE patients show a higher incidence of LN than Caucasian SLE patients and often present with more severe disease [2]. LN is also the most common form of secondary glomerulonephritis in China [3], and leads to a heavy disease burden. Improved treatments for LN patients have reduced the mortality from renal failure in recent decades [4], but the decline in mortality is counter-balanced by increased mortality and morbidity from cardiovascular disease (CVD) [5].

It has been reported that decreased HDL-C levels are common in both SLE [6] and chronic kidney disease (CKD) [7] patients. HDL-C is considered protective against atherosclerosis [8]. Strong evidence suggests that low HDL-C levels increase the risk of CVD morbidity and mortality [9, 10]. Furthermore, an increasing number of clinical studies suggest that low HDL-C levels are associated with increased risk of renal dysfunction [11–13]. In SLE patients, altered HDL-C composition induced by systemic inflammation is associated with reduced anti-inflammatory and antioxidant activity [14]. HDL-C has also been shown to be dysfunctional in CKD patients [15, 16]. What’s more, it was reported that the predictive role of HDL-C was modulated by renal function, with adverse role of high HDL-C in patients with renal dysfunction [11, 17]. As a result, although high HDL-C is usually considered protective, an increasing number of studies have revealed adverse effects of high HDL-C, with increased risk of CKD progression [11] and CVD mortality [17].

However, previous studies have mostly been in Caucasians, who differ from Asians in their genetics, lifestyles and cultures. Additionally, studies have focused on the role of HDL-C in general populations or CKD patients, rather than in LN patients. To the best of our knowledge, few data has explored the role of HDL-C levels in the prognosis of LN patients. We therefore conducted this study to evaluate the effect of HDL-C levels on the progression of LN.

## Methods

### Study patients

All patients registered in the LN database of the First Affiliated Hospital of Sun Yat-sen University between 1 January 2006 and 31 December 2011 enrolled for this study. Each study patient satisfied the criteria for SLE revised in 1997 by the American College of Rheumatology [18]. The study exclusion criteria were as follows: (1) age < 14 years; (2) drug-induced SLE; (3) malignant tumor; (4) the absence of serum HDL-C data; (5) the absence of follow-up data or follow-up time shorter than 3 months; (6) end-stage renal disease (ESRD) on admission. For each patient, a physical examination was performed and laboratory tests and renal biopsy items were recorded. Disease activity was estimated using the Systemic Lupus Erythematosus Disease Activity Index (SLEDAI) [19]. This study complied with the ethical principles outlined in the Helsinki Declaration, and the Human Ethics Committees of Sun Yat-sen University reviewed and approved the study protocol. All the participants provided signed written informed consent prior to enrollment.

### Laboratory examinations

Laboratory tests were performed at the time of diagnosis of LN. Blood samples for a complete blood count, serum creatinine, lipid profiles, antibodies were obtained after overnight fasting. Urinary sediment analysis and 24-h proteinuria were also performed at the time of diagnosis of LN. The estimated glomerular filtration rate (eGFR) was calculated using the CKD-EPI equation. Hypertension was defined as systolic blood pressure ≥ 140 mmHg and/or diastolic blood pressure ≥ 90 mmHg or treatment with antihypertensive drugs. Hypoalbuminemia was defined as serum albumin <30 g/L. Additionally, renal biopsies were investigated for glomerular damage and tubulointerstitial injury. The definition of HDL-C category followed the guidelines of the National Cholesterol Education Program Adult Treatment Panel III (NCEP ATP III): low HDL-C was defined as HDL-C level < 40 mg/dL (1.04 mmol/L) and high HDL-C was defined as HDL-C level ≥ 60 mg/dL (l.55 mmol/L) [20].

### The study outcomes

Patients were required to return to our center at least once a year for an overall medical evaluation and/or were interviewed annually by telephone by trained doctors to assess the general conditions. We defined our outcomes as follows: the primary outcome was ESRD (eGFR <15 ml/min/1.73m^2^ or entry in dialysis or renal transplantation). The secondary outcomes were all-cause mortality and CVD mortality. Causes of CVD mortality included acute myocardial infarction, atherosclerotic heart disease, cardiomyopathy, cardiac arrhythmia, cardiac arrest, congestive heart failure, cerebrovascular accident (including intracranial hemorrhage), ischemic brain damage, anoxic encephalopathy, and peripheral vascular disease. If the patients died in our hospital, the exact cause of death was recorded, and if death occurred outside our hospital, doctors obtained the cause of death from descriptions provided by family members. All patients were followed up until ESRD, death or censoring on 31 December 2012.

### Statistical analysis

HDL-C was categorized by means of the following criteria: low HDL-C when <1.04 mmol/L, intermediate HDL-C from 1.04 to <1.55 mmol/L, and high as greater than or equal to 1.55 mmol/L. Quantitative variables were shown as mean and standard deviations and were compared by ANOVA or Wilcoxon rank sum test. Qualitative variables were described by frequency and were compared by chi-square tests or Wilcoxon rank sum test. Logistic regression techniques were used to determine the risk factors associated with low and high HDL-C. The significant variates with *P* value <0.10 in univariate analysis were forced into the multivariate models. Other variates were selected into multivariable models using the forward method (entry: 0.1, removal: 0.2). Kaplan–Meier survival curves were generated to calculate the cumulative event-free survival rates and a log-rank test was used to compare the survival differences between the three groups. Cox regression models were used to assess the relationships of HDL-C categories and continuous HDL-C levels with ESRD, all-cause mortality and CVD mortality. For each analysis, three models were built based on the level of multivariable adjustment. Model 1 included age and gender. Model 2 included model 1 covariates and significant variables with *P* value <0.10 in univariate analysis forced into the model and other variables selected into the multivariate model by the forward method (entry: 0.1; removal: 0.2). Model 3 adjusted for model 2 covariates, other lipid parameters including total cholesterol, triglyceride and LDL-C and other important clinical factors. Subgroup analyses were performed to examine the effect of HDL-C levels on ESRD and all-cause mortality in patients stratified into two groups of eGFR (eGFR ≥60 ml/min/1.73m^2^ and eGFR <60 ml/min/1.73m^2^). The results of these regression analyses were presented as hazard ratio (HR) with 95% confidence intervals (95% CI). The interaction between eGFR category and serum HDL-C level on ESRD and all-cause mortality was examined by performing a formal test of interaction. A two-tailed *P*-value <0.05 was considered statistically significant. All analyses were performed with SPSS version 13.0.

## Results

### Baseline characteristics and comparisons among groups with low, intermediate and high HDL-C levels in LN patients

**Fig. 1:**
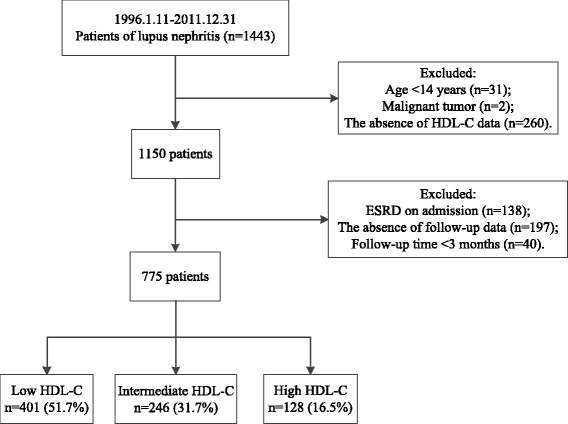
Enrollment flow chart of the study

A total of 775 patients were finally analyzed in this study (Fig. 1). Overall, the mean age was 31.2 ± 13.1 years and 645 (83.2%) were female. Mean serum HDL-C was 1.09 ± 0.49 mmol/L. The median values of eGFR, SLEDAI and 24-h urine protein were 81.8 ml/min/1.73m^2^, 14 and 1.7 g/d, respectively. 96.9% of the patients were using prednisone and 60.4% were receiving immunosuppressive treatment. 401 (51.7%) patients had low HDL-C levels and 128 (16.5%) had high HDL-C levels. The baseline characteristics of the total LN patients and the comparisons among groups with low, intermediate and high HDL-C levels were shown in Table 1. The patients with low HDL-C levels showed shorter disease duration (*P* < 0.001), lower eGFR (*P* < 0.001), higher SLEDAI (*P* < 0.001), lower total cholesterol (*P* < 0.001), higher triglyceride (*P* < 0.001), lower LDL-C (*P* < 0.001), higher incidence of hypoalbuminemia (*P* = 0.038). The patients with low HDL-C levels demonstrated a higher incidence of immunosuppressive treatment (*P* = 0.023). In addition, comparisons of baseline characteristics between LN patients with and without follow-up data were shown in Additional file 3: Table S1. LN patients without follow-up data showed longer disease duration (*P* < 0.001), lower eGFR (*P* = 0.019) and higher incidence of diabetes mellitus (*P* = 0.020).

**Table 1:** Baseline characteristics of total LN patients and comparisons among low, intermediate and high HDL-C groups

Parameters	Total (*n* = 775)	Low HDL-C (*n* = 401)	Intermediate HDL-C (*n* = 246)	High HDL-C (*n* = 128)	*P*
Age, years	31.2 ± 13.1	30.9 ± 13.6	31.8 ± 12.7	31.3 ± 12.2	0.666
Gender (female %)	645 (83.2)	328(81.8)	208 (84.6)	109 (85.2)	0.538
Smoking (%)	23 (3.0)	16 (4.0)	5 (2.0)	2 (1.6)	0.201
Weight, kg	53.8 ± 10.3	53.8 ± 11.2	53.3 ± 9.2	54.3 ± 9.3	0.675
Disease duration, months	3 (1, 16)	2 (1, 11)	6 (1, 32)	4 (1, 21)	<0.001
eGFR, ml/min/1.73m^2^	81.8 (46.2, 125.0)	71.5 (38.5, 113.3)	87.5 (52.9, 127.0)	109.9 (67.6, 135.6)	<0.001
24-h proteinuria, g	1.7 (0.8, 3.2)	1.7 (0.8, 3.3)	1.6 (0.8, 3.2)	1.8 (0.7, 3.1)	0.957
Total cholesterol, mmol/L	6.11 ± 2.47	5.52 ± 2.42	6.53 ± 2.36	7.12 ± 2.34	<0.001
Triglyceride, mmol/L	2.50 ± 1.86	2.90 ± 2.12	2.15 ± 1.38	1.89 ± 1.45	<0.001
HDL-C, mmol/L	1.09 ± 0.49	0.72 ± 0.18	1.27 ± 0.14	1.92 ± 0.39	<0.001
LDL-C, mmol/L	3.59 ± 1.82	3.13 ± 1.66	3.99 ± 1.79	4.24 ± 1.96	<0.001
SLEDAI	14 (11, 18)	16 (12, 18)	14 (10, 18)	12 (10, 16)	<0.001
Diabetes mellitus (%)	55 (7.1)	31 (7.7)	21 (8.5)	3 (2.3)	0.067
Hypertension (%)	307 (39.6)	159 (39.7)	102 (41.5)	46 (35.9)	0.584
Hypoalbuminemia	471 (60.8)	261 (65.1)	139(56.5)	71(55.5)	0.038
Global sclerosis^a^ (%)	224 (42.3)	105 (38.6)	79 (47.3)	40 (44.0)	0.188
Crescents^a^ (%)	275 (51.9)	144 (52.9)	84(50.3)	47(51.6)	0.864
Interstitial inflammation^a^ (%)	390 (73.6)	211(77.6)	115(68.9)	64(70.3)	0.098
Tubular atrophy^a^(%)	301 (56.8)	152 (55.9)	100 (59.9)	49 (53.8)	0.588
Interstitial fibrosis^a^(%)	307 (57.9)	153 (56.3)	100 (59.9)	54 (59.3)	0.723
Corticosteroid treatment (%)	751 (96.9)	387 (96.5)	240 (97.6)	124 (96.9)	0.755
Immunosuppressive treatment (%)	468 (60.4)	241 (60.1)	137 (55.7)	90 (70.3)	0.023
Lipid-lowering treatment (%)	163 (21.0)	81 (20.2)	53 (21.5)	29 (22.7)	0.815

Note: Data are presented as mean ± SD, median (25th, 75th) or number (%). ^a^Results are for the subset of the cohort for which patients had renal biopsy data (*n* = 530)

*SLEDAI* Systemic lupus erythematosus disease activity index

### Risk factors for low and high HDL-C levels

Table 2 lists significant risk factors for patients with low and high HDL-C levels by adjusting for the covariates listed in Table 1 using the enter and forward selection procedure. Low HDL-C was associated with disease duration (OR = 0.99, 95% CI 0.99–1.00, *P* = 0.010), eGFR (OR = 0.96, 95% CI 0.92–0.99, *P* = 0.018), total cholesterol (OR = 0.59, 95% CI 0.47–0.75, *P* < 0.001) and triglyceride (OR = 1.89, 95% CI 1.53–2.32, *P* < 0.001). High HDL-C was associated with total cholesterol (OR = 1.59, 95% CI 1.20–2.10, *P* = 0.001), triglyceride (OR = 0.57, 95% CI 0.42–0.77, *P* < 0.001) and immunosuppressive treatment (OR = 1.90, 95% CI 1.13–3.20, *P* = 0.016).

**Table 2:** Multivariate logistic regression models for the risk factors of low HDL-C and high HDL-C

Risk factors	OR	95% CI	*P*
Low HDL-C (n = 401)			
Disease duration, months	0.99	0.99–1.00	0.010
eGFR, 10 ml/min/1.73m^2^	0.96	0.92–0.99	0.018
Total cholesterol, mmol/L	0.59	0.47–0.75	<0.001
Triglyceride, mmol/L	1.89	1.53–2.32	<0.001
LDL-C, mmol/L	1.17	0.89–1.55	0.260
SLEDAI	1.02	0.98–1.05	0.438
Hypoalbuminemia	1.41	0.91–2.18	0.121
High HDL-C (*n* = 128)			
Total cholesterol, mmol/L	1.59	1.20–2.10	0.001
Triglyceride, mmol/L	0.57	0.42–0.77	<0.001
Immunosuppressive treatment	1.90	1.13–3.20	0.016

Note: Reference category is intermediate HDL-C (*n* = 246). The significant variates in univariate analysis were forced into the multivariate models and other variables excluding HDL-C levels listed in Table 1 were selected into the multivariate models by the forward method (entry: 0.1, removal: 0.2). Biopsy data were not included in the model

OR: odds ratio; 95% CI: 95% confidence interval. *SLEDAI* Systemic lupus erythematosus disease activity index

### HDL-C and ESRD

During the median follow-up period of 56 months (3–206 months), 71 patients (9.2%) had ESRD and 84 (10.8%) deaths occurred, 17 (20.2%) of which were associated with CVD. In Kaplan-Meier survival analysis, there was no significant difference among three HDL-C groups for ESRD (shown in Additional file 1: Figure S1). In multivariate Cox regression analysis, we found no statistical significance for the association of HDL-C category or continuous HDL-C levels with ESRD in the total cohort (Table 3).

**Table 3:** Risk of ESRD and all-cause mortality by HDL-C category and each 0.1 mmol increase of HDL-C

	Model 1		Model 2		Model 3	
	HR (95% CI)	*P*	HR (95% CI)	*P*	HR (95% CI)	*P*
ESRD						
Low HDL-C	1.01 (0.61, 1.68)	0.958	0.93 (0.48, 1.80)	0.828^a^	0.93 (0.46, 1.90)	0.847
Intermediate HDL-C	Ref	–	Ref	–	Ref	–
High HDL-C	0.64 (0.29, 1.41)	0.266	1.06 (0.40, 2.82)	0.902^a^	0.97 (0.35, 2.68)	0.959
Per 0.1 mmol/L increase	0.98 (0.93, 1.03)	0.368	1.02 (0.95, 1.09)	0.551^a^	1.01 (0.94, 1.10)	0.743
All-cause mortality						
Low HDL-C	1.94 (1.17, 3.20)	0.010	2.21 (1.13, 4.35)	0.021^b^	2.16 (1.06, 4.40)	0.033
Intermediate HDL-C	Ref	–	Ref	–	Ref	–
High HDL-C	0.51 (0.19, 1.35)	0.172	0.58 (0.16, 2.10)	0.406^b^	0.62 (0.17, 2.26)	0.464
Per 0.1 mmol/L increase	0.90 (0.85, 0.95)	<0.001	0.88 (0.82, 0.95)	0.001^b^	0.88 (0.81, 0.96)	0.005

Note: Model 1 Adjusted for age and gender. Model 2: ^a^Adjusted for age, gender, disease duration, weight, eGFR, 24-h proteinuria, hypertension and lipid-lowering therapy. ^b^Adjusted for age, gender, disease duration, weight, eGFR and hypertension. Model 3 Adjusted for age, gender, weight, eGFR, smoking status, SLEDAI, 24-h proteinuria, total cholesterol, triglyceride, LDL-C, diabetes mellitus, hypertension, hypoalbuminemia, disease duration, lipid-lowering therapy and immunosuppressive treatment

*HR* hazard ratio; 95% *CI* 95% confidence interval

We performed subgroup analyses to examine the effect of HDL-C levels on ESRD in patients stratified into two groups of eGFR. After adjustment by age, gender, weight, smoking status, eGFR, SLEDAI, 24-h proteinuria, total cholesterol, triglyceride, LDL-C, diabetes mellitus, hypertension, hypoalbuminemia, disease duration, lipid-lowering therapy and immunosuppressive treatment, with each 0.1 mmol/L increase in HDL-C level, adjusted HRs for ESRD were 0.92 (95% CI: 0.83–1.04, *P* = 0.173) for eGFR ≥60 ml/min/1.73m^2^ and 1.11 (95% CI: 1.01–1.23, *P* = 0.036) for eGFR <60 ml/min/1.73m^2^. Further, we tested the interaction between eGFR category and serum HDL-C levels on ESRD in the total cohort and found a significant interaction (β = −1.738, *P* = 0.005) (Table 4).

**Table 4:** Risk of ESRD and all-cause mortality with each 0.1 mmol/L increase of HDL-C levels by eGFR

	eGFR ≥60 ml/min/1.73m^2^ (*n* = 518)		eGFR < 60 ml/min/1.73m^2^ (*n* = 257)		eGFR category * HDL-C Interaction	
	HR (95% CI)	*P* ^a^	HR (95% CI)	*P* ^a^	β	*P* ^b^
ESRD	0.92 (0.83, 1.04)	0.173	1.11 (1.01, 1.23)	0.036	−1.738	0.005
All-cause mortality	0.86 (0.75, 0.98)	0.021	0.96 (0.87, 1.06)	0.429	−1.156	0.091

Note: ^a^Adjusted for age, gender, weight, smoking status, eGFR, SLEDAI, 24-h proteinuria, total cholesterol, triglyceride, LDL-C, diabetes mellitus, hypertension, hypoalbuminemia, disease duration, lipid-lowering therapy and immunosuppressive treatment. ^b^Adjusted for age, gender, weight, smoking status, SLEDAI, 24-h proteinuria, total cholesterol, triglyceride, LDL-C, diabetes mellitus, hypertension, hypoalbuminemia, disease duration, lipid-lowering therapy and immunosuppressive treatment. *Interactions between eGFR category and HDL-C levels

*HR* hazard ratio; 95% *CI* 95% confidence interval

### HDL-C and all-cause mortality

**Fig. 2:**
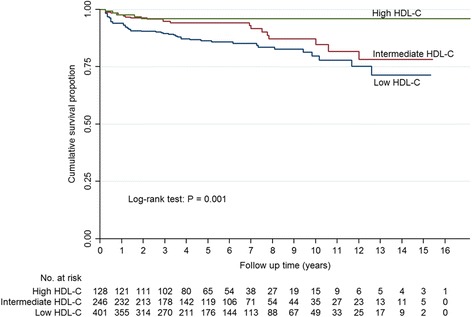
Kaplan-Meier survival curves for all-cause mortality in three HDL-C groups of LN patients. Log-rank test of three HDL-C groups: *P* = 0.001. Log-rank test of high HDL-C vs. intermediate HDL-C: *P* = 0.126; high HDL-C vs. low HDL-C: *P* = 0.002; intermediate HDL-C vs. low HDL-C: *P* = 0.017. A two-tailed *P*-value <0.05/3 was considered statistically significant

The Kaplan-Meier survival curves of the three HDL-C groups for all-cause mortality were presented in Fig. 2. There were statistically significant differences among the three HDL-C groups (log rank *P* = 0.001), with worse survival in the group with a low HDL-C level.

The result of multivariate Cox regression models for the risk of all-cause mortality by HDL-C category (with intermediate HDL-C as the reference category) and each 0.1 mmol/L increase in serum HDL-C level is presented in Table 3. Regardless of the adjustment method used, the low HDL-C group was significantly associated with increased risk of all-cause mortality compared to the intermediate HDL-C group. The risk of all-cause mortality in the high HDL-C group did not differ significantly from that of the intermediate HDL-C group. In model 3, which was a maximally adjusted model including age, gender, weight, smoking status, eGFR, SLEDAI, 24-h proteinuria, total cholesterol, triglyceride, LDL-C, diabetes mellitus, hypertension, hypoalbuminemia, disease duration, lipid-lowering therapy and immunosuppressive treatment, low HDL-C showed an adjusted HR of 2.16 (95% CI: 1.06–4.40, *P* = 0.033) for all-cause mortality. With each 0.1 mmol/L increase in HDL-C level, adjusted HR for all-cause mortality was 0.88 (95% CI: 0.81–0.96, *P* = 0.005).

We also performed subgroup analyses and interaction test to examine the effect of HDL-C levels on all-cause mortality in patients stratified into two categories of eGFR. There was no statistical significance in the interaction between eGFR categories and HDL-C, with the interaction *P* value was 0.091 (Table 4).

### HDL-C and CVD mortality

In Kaplan-Meier survival analysis, there was no significant difference among three HDL-C groups for CVD mortality (shown in Additional file 2: Figure S2). In Cox regression analysis, with age and gender as covariates, low HDL-C showed an adjusted HR of 4.77 (95% CI: 1.35–16.94, *P* = 0.016) for CVD mortality. With each 0.1 mmol/L increase in HDL-C level, adjusted HR for CVD mortality was 0.75 (95% CI: 0.58–0.97, *P* = 0.028) (shown in Additional file 3: Table S2). As there were only 17 CVD mortalities in the total cohort, we didn’t perform further adjustment and the interaction analysis.

## Discussion

In a longitudinal large cohort in one clinical research center, high HDL-C levels were associated with increased risk of ESRD in patients with advanced reduced renal function (eGFR <60 ml/min/1.73m^2^), but were not associated with ESRD in patients with reserved renal function (eGFR ≥60 ml/min/1.73m^2^). While low HDL-C levels were independently associated with all-cause mortality of LN patients. To the best of our knowledge, this cohort study is the largest ever performed to investigate the effect of HDL-C levels on prognosis of LN patients.

We found an association between decreased HDL-C level and all-cause mortality of LN patients. It is generally accepted that normal HDL-C has important physiological functions. It is the key vehicle of reverse cholesterol transport (RCT), by which cholesterol is removed from peripheral tissues, carried in the plasma, and disposed of in the liver [7]. HDL-C also have additional protective functions, including antioxidant and anti-inflammatory activity, prevention of endothelial cell apoptosis and antithrombotic effects [21]. Clinical evidence shows that low HDL-C levels are associated with increased risk of CVD after correction for other risk factors in multivariate analysis [9, 22]. Prospective studies showed that low HDL-C is usually the lipid risk factor most highly associated with CVD risk [10, 23].

However, in our study, as the number of CVD mortalities was not enough for further adjustment, we only examined the role of HLD-C levels on CVD mortality adjusted by age and gender. We found that after adjustment by age and gender, low HDL-C was associated with increased risk of CVD mortality. And with the increase in HDL-C level, the risk of CVD mortality decreased. Further studies with more CVD mortality events are needed to confirm the role of HDL-C on CVD mortality in LN patients.

It is interesting to note that our study found that the role of high HDL-C in renal outcome differs between LN patients with early and advanced renal dysfunction, which has not been reported before. The association between HDL-C and renal outcome was not statistically significant in patients with reserved renal function. It seems that the role of HDL-C was modulated by renal function. In fact, an increasing number of human and animal studies have raised the question of whether HDL-C, which is generally thought to be protective, is “good” in all situations. The US cohort of veterans showed a U-shaped relationship between HDL-C levels and renal outcomes. The risk was increased in the lowest and highest deciles of HDL-C [11]. A 3-year cohort of 33,109 chronic hemodialysis patients also showed the adverse role of high HDL-C in CVD mortality [17]. Zewinger et al. reported that associations of HDL-C with CVD and all-cause mortality are modified by eGFR. High HDL-C levels were not associated with reduced mortality risk in patients with reduced kidney function, similar to our results [24]. Additionally, pharmaceutical or genetic interventions to increase serum HDL-C levels have shown no benefit so far [25–27].

The mechanism of the adverse role of high HDL-C in patients with advanced renal dysfunction is presently unknown. Experimental evidence suggests that high HDL-C might have an adverse effect under some circumstances, including CKD. In the presence of oxidative stress and inflammation, HDL-C transforms from an antioxidant and anti-inflammatory particle to a pro-oxidant, pro-inflammatory particle known as acute-phase HDL-C [28–30], suggesting a loss of protective effect. Patients with renal dysfunction tend to have alterations not only in quantity [31], but also quality of HDL-C [15, 32]. HDL-C may not only be dysfunctional but also may promote inflammation in patients with advanced renal dysfunction [33]. Honda et al. have recently shown that dysfunctional HDL-C is produced under oxidative stress in hemodialysis patients, and this dysfunctional high HDL-C is related to increased oxidized HDL-C, which has lost its protective role and can even result in increased CVD mortality [34]. It has also been reported that HDL-C in patients with advanced renal dysfunction loses its anti-inflammatory property and even promotes production of inflammatory cytokines, when compared with HDL-C isolated from healthy controls [35, 36]. In addition, abnormal HDL-C can contribute to endothelial dysfunction and innate immunity via activation of Toll-like receptor-2 in CKD patients [37]. Overall, the reason for the adverse role of high HDL-C is still unknown and requires further study. As the structure and subpopulations of HDL-C were not measured in our study, we could not distinguish the actual particles of HDL-C that resulted in worse renal outcomes.

No significant association of low HDL-C with ESRD was found in our total cohort or in the different eGFR categories. Previous studies to examine the role of low HDL-C in renal outcome showed inconsistent results. Some studies showed that low HDL-C levels were correlated with the progression of kidney disease in the general population [38, 39] and in CKD patients [12, 13, 40], while others did not find this result [41, 42]. The Chronic Renal Insufficiency Cohort (CRIC) study of 3939 CKD patients found that low HDL-C levels were not associated with progression of CKD, which is consistent with our results [42].

A plausible explanation is that the association of low HDL-C levels with adverse renal outcome in previous studies may not be causal in nature. Low HDL-C might merely be a marker of an adverse metabolic situation, including severe inflammation and oxidative stress, which are common in SLE and CKD patients [14, 42]. The existence of these factors might partially or fully explain the observed association in previous studies. This interpretation is supported by observations from a post-hoc analysis of the AIM-HIGH trial [43]. Compared with the placebo group, CKD participants receiving niacin had a significant increase in HDL-C, but the addition of niacin did not improve CVD outcomes or renal function, and was even associated with a higher risk of all-cause mortality.

We also compared baseline characteristics of patients who were lost to follow up with the included patients in this study. LN patients without follow-up data showed longer disease duration, worse renal function and higher incidence of diabetes mellitus. And there was no significant difference between two groups in other items. These three factors were further adjusted in the multivariate Cox regression models to reduce potential confounders. So the exclusion of the patients without follow-up data may not affect the results and conclusion of the study.

The strengths of our study are as follows: (1) this prospective cohort study included a large number of LN patients with a long follow-up period in a single center, (2) the association of HDL-C levels with outcomes was examined with adjustments for demographics, laboratory items and treatment factors. The study also has some limitations. First, we only collected single baseline lipid levels for analysis, from which we could not evaluate the development and progression of lipid abnormalities. What’s more, only HDL-cholesterol was measured. HDL structure and subpopulation distribution changes, which may also contribute to the increased mortality of LN patients, were not detected in current study. Second, in multivariate Cox regression models analysis, the role of HLD-C levels on CVD mortality were only adjusted by age and gender. As the number of CVD mortality is not enough for further adjustment, we didn’t include other covariates for adjustment.

## Conclusions

In conclusion, high HDL-C levels were associated with increased risk of ESRD in patients with advanced renal dysfunction, while low HDL-C levels were associated with all-cause mortality. Further studies will be necessary to find the optimal HDL-C levels and to examine the changes of HDL structure and subpopulations distribution in LN patients.

## Additional files

Additional file 1: Figure S1. Kaplan-Meier survival curves for ESRD in three HDL-C groups of LN patients. There were no significant differences among the low, intermediate and high groups (*P* = 0.459). (DOCX 75 kb)

Additional file 2: Figure S2. Kaplan-Meier survival curves for CVD mortality in three HDL-C groups of LN patients. There were no significant differences among the low, intermediate and high groups (*P* = 0.489). (DOCX 73 kb)

Additional file 3: Table S1. Comparisons of baseline characteristics between LN patients with and without follow-up data. **Table S2.** Risk of CVD mortality by HDL-C category and each 0.1 mmol/L increase of HDL-C levels. (DOCX 78 kb)

## Abbreviations

CKD: Chronic kidney disease
CVD: Cardiovascular disease
ESRD: End-stage renal disease
HDL-C: High density lipoprotein cholesterol
HR: Hazard ratio
LDL-C: Low density lipoprotein cholesterol
LN: Lupus nephritis
NCEP ATP III: National cholesterol education program adult treatment panel III
OR: Odds ratio
SLE: Systemic lupus erythematosus
SLEDAI: Systemic lupus erythematosus disease activity index
