# Supplementary figures and images for: Effect of low and high HDL-C levels on the prognosis of lupus nephritis patients: a prospective cohort study
Source: Lipids Health Dis. 2017 Dec 6;16:232. doi: 10.1186/s12944-017-0622-3 (PMC5719733; doi:10.1186/s12944-017-0622-3)

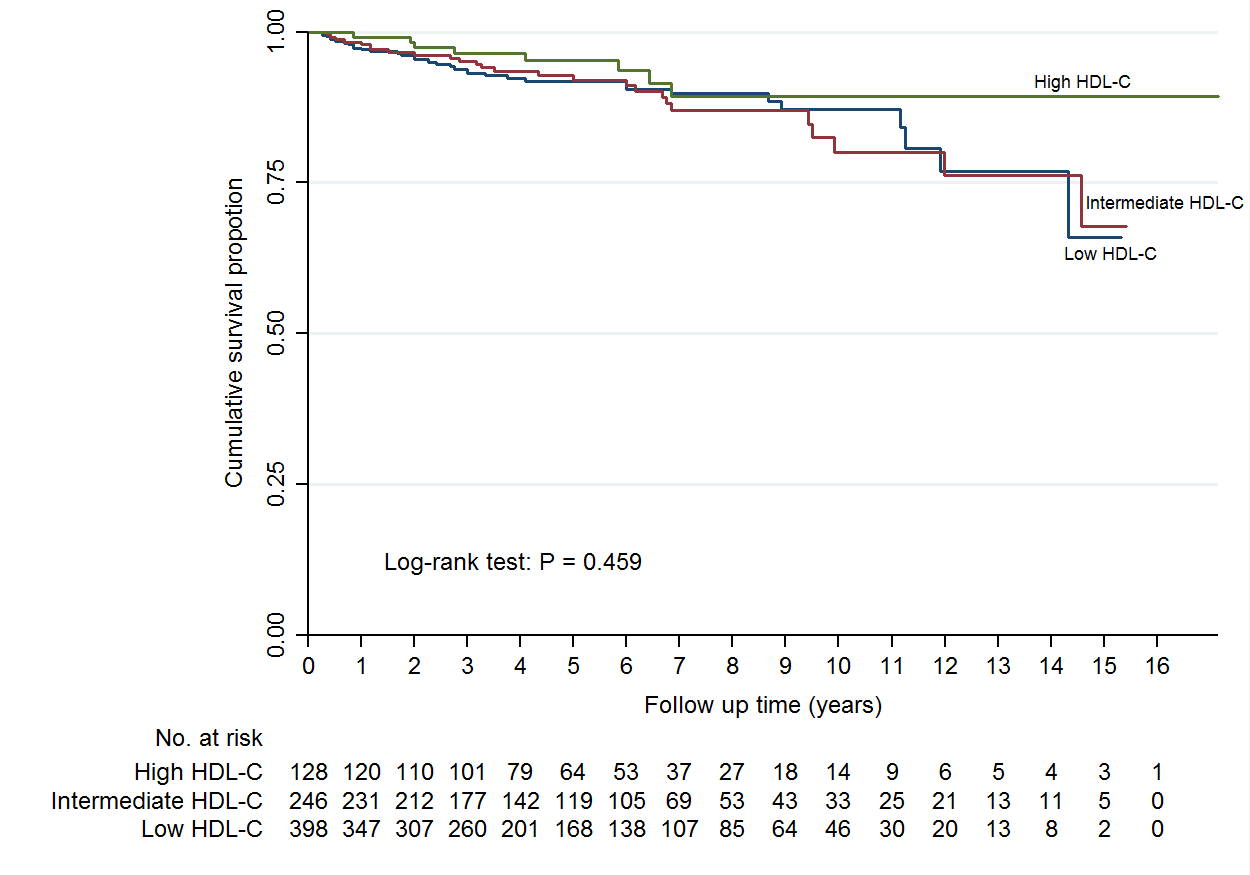

Supplement: Supplementary file 1 — Kaplan-Meier survival curves for ESRD in three HDL-C groups of LN patients. There were no significant differences among the low, intermediate and high groups (P = 0.459). (DOCX 75 kb) [file 12944_2017_622_MOESM1_ESM.docx]

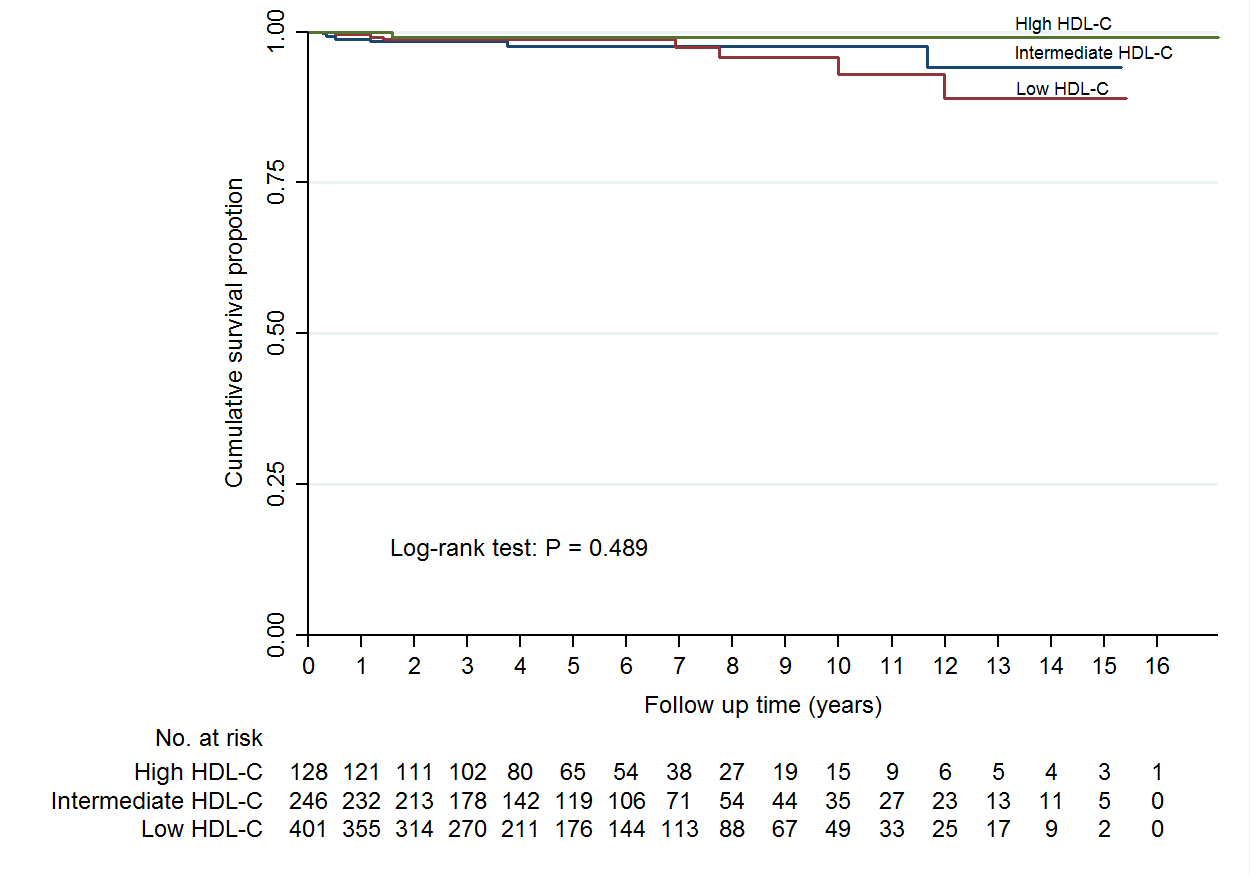

Supplement: Supplementary file 2 — Kaplan-Meier survival curves for CVD mortality in three HDL-C groups of LN patients. There were no significant differences among the low, intermediate and high groups (P = 0.489). (DOCX 73 kb) [file 12944_2017_622_MOESM2_ESM.docx]
