# Supplementary material for: Effect of low and high HDL-C levels on the prognosis of lupus nephritis patients: a prospective cohort study
Source: Lipids Health Dis. 2017 Dec 6;16:232. doi: 10.1186/s12944-017-0622-3 (PMC5719733; doi:10.1186/s12944-017-0622-3)
Supplement: Supplementary file 3 — Comparisons of baseline characteristics between LN patients with and without follow-up data. Table S2. Risk of CVD mortality by HDL-C category and each 0.1 mmol/L increase of HDL-C levels. (DOCX 78 kb) [file 12944_2017_622_MOESM3_ESM.docx]

**Table S1** Comparisons of baseline characteristics between LN patients with and without follow-up data

| Parameters | With follow-up data (n = 775) | Without follow-up data (n = 197) | *P* |
| --- | --- | --- | --- |
| Age, years | 31.2±13.1 | 30.6±12.1 | 0.574 |
| Gender (female %) | 645 (83.2) | 157(79.7) | 0.244 |
| Smoking (%) | 23 (3.0) | 6 (3.0) | 0.954 |
| Weight, kg | 53.8±10.3 | 53.2±10.2 | 0.535 |
| Disease duration, months | 3 (1, 16) | 18 (4, 37) | <0.001 |
| eGFR, ml/min/1.73m^2^ | 81.8 (46.2, 125.0) | 76.2 (40.3, 106.6) | 0.019 |
| 24-h proteinuria, g | 1.7 (0.8, 3.2) | 1.4 (0.7, 2.6) | 0.092 |
| Total cholesterol, mmol/L | 6.11±2.47 | 6.02±2.42 | 0.662 |
| Triglyceride, mmol/L | 2.50±1.86 | 2.53±1.99 | 0.833 |
| HDL-C, mmol/L | 1.09±0.49 | 1.15±0.61 | 0.221 |
| LDL-C, mmol/L | 3.59±1.82 | 3.50±1.77 | 0.539 |
| SLEDAI | 14 (11, 18) | 14 (10, 18) | 0.962 |
| Diabetes mellitus (%) | 55 (7.1) | 24 (12.2) | 0.020 |
| Hypertension (%) | 307 (39.6) | 75 (38.1) | 0.692 |
| Hypoalbuminemia | 471 (60.8) | 119 (60.4) | 0.925 |
| Global sclerosis ^a^ (%) | 224 (42.3) | 54 (38.6) | 0.430 |
| Crescents^a^ (%) | 275 (51.9) | 68 (48.6) | 0.485 |
| Interstitial inflammation ^a^ (%) | 390 (73.6) | 102 (72.9) | 0.862 |
| Tubular atrophy ^a^ (%) | 301 (56.8) | 73 (52.1) | 0.324 |
| Interstitial fibrosis ^a^ (%) | 307 (57.9) | 76 (54.3) | 0.439 |
| Corticosteroid treatment (%) | 751 (96.9) | 191 (97.0) | 0.970 |
| Immunosuppressive treatment (%) | 468 (60.4) | 118 (59.9) | 0.900 |
| Lipid-lowering treatment (%) | 163 (21.0) | 31 (15.7) | 0.097 |

Note: Data are presented as mean ± SD, median (25th, 75th) or number (%). ^a^Results are for the subset of the cohort for which patients had renal biopsy data (n=530 in group with follow-up data and n=140 in group without follow-up data)

SLEDAI: Systemic Lupus Erythematosus Disease Activity Index.

**Table S2** Risk of CVD mortality by HDL-C category and each 0.1mmol/L increase of HDL-C levels

|  | Model 1 | | Model 2 | | Model 3 | |
| --- | --- | --- | --- | --- | --- | --- |
|  | HR (95% CI) | P | HR (95% CI) | P | HR (95% CI) | P |
| **CVD mortality** |  |  |  |  |  |  |
| Low HDL-C | 4.77 (1.35, 16.94) | 0.016 | — | — | — | — |
| Intermediate HDL-C | Ref | — | — | — | — | — |
| High HDL-C | 15.60 (0.86, 283.58) | 0.063 | — | — | — | — |
| Per 0.1mmol/L increase | 0.75 (0.58, 0.97) | 0.028 | — | — | — | — |

Note: Model 1 Adjusted for age and gender. Further adjustment in model 2 and model 3 was not applicable as the number of CVD mortality endpoint is not enough for analysis.

HR: hazard ratio; 95% CI: 95% confidence interval.
